# Supplementary material for: The interaction of relative age with maturation and body size in female handball talent selection
Source: PLoS One. 2024 Oct 21;19(10):e0298560. doi: 10.1371/journal.pone.0298560 (PMC11493251; doi:10.1371/journal.pone.0298560)
Supplement: Attachment — Submitted filename: Review 1.pdf [file pone.0298560.s002.pdf]

Specific and general comments (by section)

ABSTRACT

- In the first lines of the abstract, the priority areas of talent identification are mentioned. However, the authors consider putting forward the idea of implementing an integrated talent identification and development system (four corners or 360º) in which all the determining factors are included, and not one above the others. Then, of course, specify in relation to the RAE and maturity status.
- Review the sentence on the study objective from a formal point of view as well as in relation to the comments made throughout the manuscript.
- Please add the average age of the players because the competition categories in international handball are of two years and the variability is large.
- The method, in terms of procedures and statistical analysis, needs to be more precise.
- The abstract should close with a powerful conclusion and, if possible, a practical application.

INTRODUCTION

- Pag. 3 (line 40). The references provided are specifically related to the RAE (systematic reviews). However, the sentence also refers to a maturity status or level. Therefore, please provide some reference to this.
- Pag. 3 (lines 35-44). In my opinion, and in relation to the previous comment, the first paragraph does not differentiate between two phenomena that have been shown to operate independently and that often tend to be confused (see Towlson, C, MacMaster, C, Parr, J & Cumming, S 2022, 'One of these things is not like the other: time to differentiate between relative age and biological maturity selection biases in soccer?', Science and Medicine in Football , vol. 6, no. 3, pp. 273-276. https://doi.org/10.1080/24733938.2021.1946133). The wording does not help to clarify both concepts and how they operate in talent detection systems.
- Pag. 3 (line 46). Reference 15 is repeated ([1]). Moreover, it and reference 16 are not useful to explain the RAE from a physical perspective.

- Pag. 3 (lines 48-50). I do not agree with this statement at all. In fact, the scientific evidence presented is limited, not considering specific handball studies by authors such as Schorer, de la Rubia, Wrang, Krahenbühl or Saavedra among others.
- Pag. 3 (line 51). The term “maturity” is not correctly used. Instead, concepts such as “maturational bias” or “maturity influence” should appear.
- Pag. 4 (lines 60-66). I agree with the authors that there is a gap in the analysis of talent identification and development processes in general, and the influence of RAE and maturity status in particular. However, there is scientific literature on some of these aspects, separately or together in a single paper:
  - Aouichaoui, C., Krichen, S., Tounsi, M., Ammar, A., Tabka, O., Chatti, S., ... & Trabelsi, Y. (2024). Reference Values of Physical Performance in Handball Players Aged 13–19 Years: Taking into Account Their Biological Maturity. Clinics and Practice, 14(1), 305-326.
  - de la Rubia, A., Kelly, A. L., García-González, J., Lorenzo, J., Mon-López, D., & Maroto-Izquierdo, S. (2023). Biological maturity vs. relative age: Independent impact on physical performance in male and female youth handball players. Biology of Sport, 41(3), 3-13.
- Pag. 4 (lines 67-72). With regard to the objective of the study, in my opinion, it should be worded differently. Neither the RAE nor the three related factors (maturation, body composition and body size) influence the chances of a player to enter the talent system in handball as this differential factor is the sport-specific tests. Moreover, if we consider the regressions performed as predictors of the selection process, the objective should be oriented in a different way by mixing both concepts.

GENERAL COMMENT: The introduction presents a structure that does not clarify the key concepts of the research topic (RAE, maturation, talent development and identification systems, etc.). Moreover, in almost all aspects it is not specific to the sport in question (handball), providing decontextualized references in some cases. This last point would be understandable if there were no scientific literature on handball, but it is abundant in some cases. In my opinion, the Introduction section should be more precise and specific.

MATERIAL AND METHODS

Study Design:

- Pag. 4 (lines 75-84). This paragraph does not provide an approach to the study design, but mixes content from the subsections Procedure and Statistical Analysis.

Sample:

- Pag. 6 (lines 123-126). Explain, as exclusion criteria, the reasons why 530 players were measured out of 3198 born in that two-year cycle.
- Pag. 7 (lines 131-132). The sample distribution into eight quartiles (odd and even year) should be stated in the text of the manuscript and not in Table 1.
- Pag. 8 (lines 150-162). The protocol for measuring the three factors (anthropometric, body composition and maturity status) is very well detailed. I congratulate the authors for calculating the maturity status through skeletal age as it is one of the most reliable procedures to determine the biological development of the participants.

The Selection Program:

- Regarding the talent programme based on only sport-specific tasks, I have three concerns: (i) given that this type of specific tasks are not carried out from phase 1 to phase 2, could it cause a selection bias? It may be that the coach's recommendation and the results of these tests are not the same so that players who are not potential talents could enter the system or vice versa according to the variables of analysis; (ii) why did the players not perform the sport-specific technical tasks in the selection process between county and regional levels; and (iii) are the tests and scores pre-validated or are they ad hoc tasks that you consider valid to assess handball performance?

RESULTS

- Pag. 10 (lines 205-210). As I interpret Figure 3, the differences in the factors indicated in relation to the RAE do not occur exclusively between Q1-Q2 and Q7-Q8. In fact, in some cases, such as biological maturity, there are no such differences between the first and last quartiles. Could the authors please specify the results in Figure 3?

- Pag. 10 (lines 224-226). Please move this exclusion criterion in the Method section.

GENERAL COMMENT: In line with the purpose of the study, the Results section lacks a mixed analysis of the three factors (maturation, body size and body composition) between the selection phase and the quartiles. These are done separately in Figures 3 and 4, however, the joint consideration of both would have made it possible to determine what happens with the three factors (6 subfactors) in each phase and according to the RAE.

DISCUSSION

- Pag. 13 (lines 254-255). This sentence is not counted correctly expressed. Perhaps it can be said that "traditional talent detection systems have inadvertently led to a prevalence of the RAE in selection processes"
- Pag. 13 (lines 254-257). The authors still do not differentiate the concepts of RAE and biological maturity. They are independent phenomena that do not have to come together at the same time. Specifically, the study by Matthys et al. It does not analyze the RAE but the maturity status. Hence, a reduction in the impact of the RAE cannot be extrapolated through the specific tasks of the sport, at least from this study.
- Pag. 14 (lines 267-269). Could the authors provide references for this statement?
- Pag. 15 (lines 294-297). This is what I was referring to in the comment on the study objective. I think that the joint analysis by phase and RAE would have provided a more global vision about the impact on the talent selection process based on specific sports tasks. Therefore, I consider that it is a limitation that must be noted.
- Pag. 15 (line 302). Krahenbühl's study talks about the perception of the coaches and their subsequent relationship with the RAE. However, the first predictor model at the county level contains maturation level and body size, which may or may not be associated with the RAE. Therefore, the argument in this regard seems imprecise to me.

GENERAL COMMENT: The Discussion presents problems, some of them already discussed in the Introduction. On the one hand, there is still no distinction between RAE

and maturity status as two different constructs. On the other hand, the references used are not valid in some cases and in parts of this section (i.e., lines 262-282) there is no reference that supports the results or explanatory arguments.
