# Supplementary material for: The interaction of relative age with maturation and body size in female handball talent selection
Source: PLoS One. 2024 Oct 21;19(10):e0298560. doi: 10.1371/journal.pone.0298560 (PMC11493251; doi:10.1371/journal.pone.0298560)
Supplement: Attachment — Submitted filename: Response to reviewers.docx [file pone.0298560.s003.docx]

**Dear Editor and Reviewers**

We would like to sincerely thank the editor and the reviewers for their time to review this manuscript and for their valuable comments, which improve the manuscript’s quality. Please find our response to the comments below. Corrections in the revised manuscript appear in Track changes function.

**Response to Reviewer 1**

Please use the space provided to explain your answers to the questions above. You may also include additional comments for the author, including concerns about dual publication, research ethics, or publication ethics. (Please upload your review as an attachment if it exceeds 20,000 characters)

Reviewer #1: I am grateful for the effort made by the authors in carrying out this scientific study, which I consider to be of relevance within the field of application of talent detection in handball.

Dear reviewer 1. Thank you for this detailed review.

However, I would like to express some of my concerns and suggestions in the comments made in the attached document, all in the interest of improving the quality of the manuscript. I hope they will be useful.

We revised the manuscript considering all the concerns raised in the review. Please find our responses for each comment separately below.

ABSTRACT

• In the first lines of the abstract, the priority areas of talent identification are mentioned. However, the authors consider putting forward the idea of implementing an integrated talent identification and development system (four corners or 360º) in which all the determining factors are included, and not one above the others. Then, of course, specify in relation to the RAE and maturity status.

We made revisions in the abstract including also the first sentence. We tried to better address the concept that relative age and maturation are two separate factors that may influence talent selection independently from each other, as this was one of the main criticisms in the review.

• Review the sentence on the study objective from a formal point of view as well as in relation to the comments made throughout the manuscript.

We understand the reviewer’s point of view regarding the analysis of maturation and body dimensions for each relative age group separately, which could be more informative about the interaction of relative age with the examined factors. We have added new results in this response (lines 260-272); however (at first place) we opted not to include them in the manuscript. As mentioned in the manuscript (lines 343-345) analysis by relative age group and selection stage is quite inconsistent making the results hard to follow. Further, comparisons between selected and not-selected players could be questioned in some cases due to the few (or none) participants in several subgroups. We rephrased the purpose of the study to align more with the conducted analysis.

• Please add the average age of the players because the competition categories in international handball are of two years and the variability is large.

Corrected accordingly for the selected and not-selected players.

• The method, in terms of procedures and statistical analysis, needs to be more precise.

We rephrased description of the methods while also considering length limitations for the abstract.

• The abstract should close with a powerful conclusion and, if possible, a practical application.

We added few sentences at the end of the abstract trying to summarize the most important findings of the study. More particularly, that (1) the RAEs were evident in this type of talent selection for female players, (2) relative age was connected to bone age, but not to stature or muscle mass, and (3) early maturation increased selection chances when selection was completed based on the coaches’ subjective evaluation, but not convincingly when sport-specific tasks were applied.

INTRODUCTION

• Pag. 3 (line 40). The references provided are specifically related to the RAE (systematic reviews). However, the sentence also refers to a maturity status or level. Therefore, please provide some reference to this.

Thank you for this comment, we added few references related to the influence of maturation on talent selection (line 48.) (Malina et al., 2015; Cumming et al., 2017; Hill et al. 2020)

# Malina RM, Rogol AD, Cumming SP, Coelho e Silva MJ, Figueiredo AJ. Biological maturation of youth athletes: assessment and implications. Br J Sports Med 2015; 49: 852–859. doi:10.1136/bjsports-2015-094623

# Cumming SP, Lloyd RS, Oliver JL, Eisenmann J, Malina RM. Bio-banding in sport: applications to competition, talent identification, and strength and conditioning of youth athletes. Strength Cond. J 2017;39 (2): 34–47 doi:[10.1519/SSC.0000000000000281](http://dx.doi.org/10.1519/SSC.0000000000000281)

# Hill M, Scott S, Malina RM, McGee D, Cumming SP. Relative age and maturation selection biases in academy football. J Sports Sci. 2020;38(11-12):1359-1367. doi:10.1080/02640414.2019.1649524.

• Pag. 3 (lines 35-44). In my opinion, and in relation to the previous comment, the first paragraph does not differentiate between two phenomena that have been shown to operate independently and that often tend to be confused (see Towlson, C, MacMaster, C, Parr, J & Cumming, S 2022, 'One of these things is not like the other: time to differentiate between relative age and biological maturity selection biases in soccer?', Science and Medicine in Football , vol. 6, no. 3, pp. 273-276. https://doi.org/10.1080/24733938.2021.1946133). The wording does not help to clarify both concepts and how they operate in talent detection systems.

We have revised the main part of the introduction trying to address more clearly the independent influence of relative age and maturation on talent selection. In the revised manuscript we refer to this in (line 50-57) and also, we discuss the related literature in separate paragraphs (Ln58-64 and Ln65-72). Moreover, we have added some new references (Sweeney et al., 2023; Towlson et al., 2022; de la Rubia et al., 2024), which describe relative age and maturation as two distinct constructs within talent selection. Although it was not intentional, we understand that initially these two factors were presented as referring to the same phenomenon resulting in confusing interpretation. Hopefully, it is clearer in the revised manuscript.

# Sweeney L, Cumming SP, MacNamara Á, Horan D. A tale of two selection biases: The independent effects of relative age and biological maturity on player selection in the Football Association of Ireland’s national talent pathway. Int J Sports Sci Coach. 2023;18(6): 1992–2003. doi:10.1177/17479541221126

# Towlson C, MacMaster C, Parr J, Cumming S. One of these things is not like the other: time to differentiate between relative age and biological maturity selection biases in soccer? Sci Med Footb. 2022;6(3): 273-276. doi: 10.1080/24733938.2021.1946133.

# de la Rubia A, Kelly AL, García-González J, Calvo JL, Mon-López D, Maroto-Izquierdo S. Biological maturity vs. relative age: Independent impact on physical performance in male and female youth handball players Biol Sport. 2024;41(3): 3–13 doi:10.5114/biolsport.2024.132999

• Pag. 3 (line 46). Reference 15 is repeated ([1]). Moreover, it and reference 16 are not useful to explain the RAE from a physical perspective.

In the revised manuscript we removed this part and accordingly the cited papers due to the changes in the structure of the introduction. After the revisions we considered that this sentence did not contribute significantly to support the rationale of the study.

• Pag. 3 (lines 48-50). I do not agree with this statement at all. In fact, the scientific evidence presented is limited, not considering specific handball studies by authors such as Schorer, de la Rubia, Wrang, Krahenbühl or Saavedra among others.

We thank the reviewer for this comment. We extended handball-specific literature in the revised manuscript discriminating the papers examining the effects of relative age (Ln65-72) and papers examining maturation (Ln58-64) on either performance metrics or on talent selection.

• Pag. 3 (line 51). The term “maturity” is not correctly used. Instead, concepts such as “maturational bias” or “maturity influence” should appear.

Corrected accordingly (changed the term maturity to maturational bias), In addition, we removed reference to the relative age from this sentence because in the cited papers (Vandendriessche et al. 2012; Matthys et al., 2012) the authors included only measures of maturation in their analysis, but not relative age.

• Pag. 4 (lines 60-66). I agree with the authors that there is a gap in the analysis of talent identification and development processes in general, and the influence of RAE and maturity status in particular. However, there is scientific literature on some of these aspects, separately or together in a single paper:

The gap in the literature refers to the lack of data for female athletes. Most studies in the literature include male athletes and as mentioned in the manuscript we consider it important to have more data from female athletes. Such an aspect may be of interest considering also that the connection of physical characteristics and body dimensions with performance metrics and talent selection seems to be more evident in male athletes and to a lesser degree in female athletes. Thus, it is interesting to examine maturation and body size in relation to talent selection in a cohort of female athletes. We added a sentence (Lines 84-85) in the revised manuscript to address this issue and also we extended handball-specific literature including also references suggested by the Reviewer (Aouichaoui et al., 2024; de la Rubia et al., 2023).

• Pag. 4 (lines 67-72). With regard to the objective of the study, in my opinion, it should be worded differently. Neither the RAE nor the three related factors (maturation, body composition and body size) influence the chances of a player to enter the talent system in handball as this differential factor is the sport-specific tests. Moreover, if we consider the regressions performed as predictors of the selection process, the objective should be oriented in a different way by mixing both concepts.

Indeed, the criteria of the examined selection program consisted of sport-specific tasks, which, as pointed, served as the differential factor. Exactly because of this type of selection, we aimed to examine whether typical factors (e.g. maturity, body dimensions) that often influence talent selection and performance potentials in young athletes have any effect on the selection. If the selection would have included (also) anthropometric and physical performance tests, then such analysis would be of less interest. We rephrased the purpose of the study to describe the nature and the content of the conducted analysis more precisely (Line 90-97).

GENERAL COMMENT:

The introduction presents a structure that does not clarify the key concepts of the research topic (RAE, maturation, talent development and identification systems, etc.). Moreover, in almost all aspects it is not specific to the sport in question (handball), providing decontextualized references in some cases. This last point would be understandable if there were no scientific literature on handball, but it is abundant in some cases. In my opinion, the Introduction section should be more precise and specific.

In the revised introduction we tried to emphasize better the distinct influence of relative age and maturity separating the relevant literature to support the rationale of this research. In addition, we extended handball-specific literature, taking into account (1) differentiation between relative age and maturity and (2) the diversified results of the relative age effects in handball research, particularly among female players.

MATERIAL AND METHODS

Study Design:

• Pag. 4 (lines 75-84). This paragraph does not provide an approach to the study design, but mixes content from the subsections Procedure and Statistical Analysis.

We rephrased most parts of the study design specifying the type of the design that we applied in this research and adding short description of the steps we followed according to the research goals. This approach consisted of three levels: first we examined the prevalence of the RAEs at the basic level of all registered players comparing the birth distribution to that of the average population. Then, to examine the effects of the selection program on the RAEs we compared birth distribution at every stage of the selection to that of all registered players in the examined age-group. We refer to this in lines 103-107. Finally, to understand the connection of maturity, body size, and body composition with the outcome of the selection we performed measurements on all selected players and on a subsample of not-selected players. This information is mentioned in lines 107-110. We removed any reference to the statistical methods from this paragraph.

Sample:

• Pag. 6 (lines 123-126). Explain, as exclusion criteria, the reasons why 530 players were measured out of 3198 born in that two-year cycle.

As also mentioned above, measurements were performed on all players who were selected to participate in the selection process and on a sub-sample of not-selected players. Measurements on all 3198 players in this age group would be more than desirable, however, this seemed extremely difficult to complete. The sub-sample of the selected players was decided by coaches and the experts of the federation, who supervised and completed the selection process. We measured all selected players. The inclusion criteria in this case were the outcome of being selected. In addition, we performed measurements on a sub-sample of not-selected players. Players were recruited based on the country region and competition level for representative purposes using a simple random sampling method. Measured players represented about 17% of all registered players in the examined age group. We have added this information under methods/participants in lines 165-172.

• Pag. 7 (lines 131-132). The sample distribution into eight quartiles (odd and even year) should be stated in the text of the manuscript and not in Table 1.

Unfortunately, we are not sure about the reviewer’s comment here. We have included in the manuscript grouping methodology based on birth quartiles (lines 173-175), however we opted to keep the presentation of each quartile’s frequency at every stage of the selection for better clarity due to the large quantity of the data. Since we generated a table containing frequency results, we considered to also include the frequency of the average population and of all registered players providing in this way a comprehensive presentation of quartile’s frequencies at every measured level and according to selected/not-selected groups. Hopefully, it can be accepted in this form.

• Pag. 8 (lines 150-162). The protocol for measuring the three factors (anthropometric, body composition and maturity status) is very well detailed. I congratulate the authors for calculating the maturity status through skeletal age as it is one of the most reliable procedures to determine the biological development of the participants.

Thank you.

The Selection Program:

• Regarding the talent programme based on only sport-specific tasks, I have three concerns: (i) given that this type of specific tasks are not carried out from phase 1 to phase 2, could it cause a selection bias? It may be that the coach's recommendation and the results of these tests are not the same so that players who are not potential talents could enter the system or vice versa according to the variables of analysis; (ii) why did the players not perform the sport-specific technical tasks in the selection process between county and regional levels; and (iii) are the tests and scores pre-validated or are they ad hoc tasks that you consider valid to assess handball performance?

First comment: indeed, the reviewer is right that there may be a selection bias between the first and the following stages due to the different selection criteria. The structure of the selection program was designed and decided by the Hungarian Handball federation, this same structure has been implemented for the last 8 years with 8 selection programs being completed in this form. Since we could not manipulate the selection, we attempted to interpret the results according to the applied criteria, we discuss the issue of coaches’ recommendation in the discussion under (Line 346-357) Of interest is to understand any possible connection between the examined factors and the selection even when selection is based on the subjective evaluation of team coaches.

Second comment: there may be a confusion here, possibly arising from the unprecise description of the selection program. We have revised the description of the selection throughout the entire manuscript including in figure 1 and in the paragraph under methods/selection program. From county to regional stage (selection 2) the players performed both generic sport-specific tasks and position-specific tasks. Indeed, from regional to national stage (selection 3), players performed only the position-specific tasks. This happened because at this stage selection was completed also according to playing positions, the experts committee considered that the generic skill of the players reaching this stage are of acceptable quality. We have included this information under methods, description of the selection program (Lines 135-161). Hopefully, the description is clear.

Third comment: again, we agree with the reviewer regarding the validity of the selection tasks. As mentioned many times, the selection tasks were designed by experts and federation coaches of the Handball Federation. It was out of our competence to evaluate these tasks, although we agree that a separate validation study would be useful, we are more than pleased to undertake this commitment in future research.

RESULTS

• Pag. 10 (lines 205-210). As I interpret Figure 3, the differences in the factors indicated in relation to the RAE do not occur exclusively between Q1-Q2 and Q7- Q8. In fact, in some cases, such as biological maturity, there are no such differences between the first and last quartiles. Could the authors please specify the results in Figure 3?

We understand the reviewer’s point. We rephrased description of the results for figure 3 attempting to provide better clarity and accuracy. Description of these results in the revised manuscript appears in (Line 242-249).

• Pag. 10 (lines 224-226). Please move this exclusion criterion in the Method section.

Corrected accordingly.

GENERAL COMMENT: In line with the purpose of the study, the Results section lacks a mixed analysis of the three factors (maturation, body size and body composition) between the selection phase and the quartiles. These are done separately in Figures 3 and 4, however, the joint consideration of both would have made it possible to determine what happens with the three factors (6 subfactors) in each phase and according to the RAE.

Yes, this is an important and interesting point. We have completed such analysis, however, we found statistical results hard to follow and quite diverse. Further, the presentation of these results for each selection stage requires 3 x 6 figures (18 figures in total), which seemed to exceed the capacity of a single manuscript. Since differences between selected and not-selected players seemed to be independent from relative age we decided to merge quartiles and emphasize selected vs not-selected comparisons in a more straightforward manner. In addition, the low number of participants in some quartile groups resulted in biased statistical comparisons. We have included these results below (figures 1-3) and certainly we are open to accept further suggestions.

**Fig 1. Boxplots of selected and not-selected players by relative age group for (A) body mass, (B) body height, (C) percent body fat, (D) muscle mass, (E) bone age and (F) maturity status at the stage 1.** *: *p*<.05 between quartiles.

**Fig 2. Boxplots of selected and not-selected players by relative age group for (A) body mass, (B) body height, (C) percent body fat, (D) muscle mass, (E) bone age and (F) maturity status at the stage 2.** *: *p*<.05 between quartiles.

**Fig 3. Boxplots of selected and not-selected players by relative age group for (A) body mass, (B) body height, (C) percent body fat, (D) muscle mass, (E) bone age and (F) maturity status at the stage 3.**

DISCUSSION

• Pag. 13 (lines 254-255). This sentence is not counted correctly expressed. Perhaps it can be said that "traditional talent detection systems have inadvertently led to a prevalence of the RAE in selection processes".

Corrected as suggested.

• Pag. 13 (lines 254-257). The authors still do not differentiate the concepts of RAE and biological maturity. They are independent phenomena that do not have to come together at the same time. Specifically, the study by Matthys et al. It does not analyze the RAE but the maturity status. Hence, a reduction in the impact of the RAE cannot be extrapolated through the specific tasks of the sport, at least from this study.

We have rephrased this sentence trying to better differentiate relative age from maturity.

• Pag. 14 (lines 267-269). Could the authors provide references for this statement?

We added separate references to support the superior body size of relative older players (Chamacho-Cardenosa et al., 2018) and their advanced maturity (Müller et al., 2017) compared to relative younger players.

• Pag. 15 (lines 294-297). This is what I was referring to in the comment on the study objective. I think that the joint analysis by phase and RAE would have provided a more global vision about the impact on the talent selection process based on specific sports tasks. Therefore, I consider that it is a limitation that must be noted.

Yes, this has been pointed also earlier during the review (and in our response), we included a sentence under limitations to address this issue. While interesting, the results suggest that differences between selected and not-selected players were independent from relative age (please see also figures 1-3 earlier in this document), thus we considered it clearer to present the results in a more comprehensive form. However, and as stated earlier, we are open to any further recommendations.

• Pag. 15 (line 302). Krahenbühl’s study talks about the perception of the coaches and their subsequent relationship with the RAE. However, the first predictor model at the county level contains maturation level and body size, which may or may not be associated with the RAE. Therefore, the argument in this regard seems imprecise to me.

Yes, we agree with the Reviewer, and we thank you for this comment. We have rephrased this sentence (Ln350-353). We also removed citation to Krahenbühl’s study, and we included a new paper (Cripps et al., 2016) which describes coaches’ perception in relation to maturation and not to relative age. Hopefully, it is more accurate in this way.

GENERAL COMMENT: The Discussion presents problems, some of them already discussed in the Introduction. On the one hand, there is still no distinction between RAE and maturity status as two different constructs. On the other hand, the references used are not valid in some cases and in parts of this section (i.e., lines 262-282) there is no reference that supports the results or explanatory arguments.

While keeping the structure of the discussion, in the revised manuscript we tried to better discriminate the two factors (maturation and relative age) interpreting the results separately. Were pointed, we added new references to support our statements (e.g. Ln315) or replaced some to be more specific (e.g. Ln350).

**Response to Reviewer 2**

Reviewer #2: PLOS One

Thank you for the opportunity to review your paper, that I read with great interest. The study is a novel contribution to the literature and the manuscript is clearly written. Your finding that technical tests cannot eliminate the impact of relative age and consequently biological development, is very interesting. Furthermore, that early maturation did not increase selection odds is equally intriguing. Following your reasoning, it would be interesting to use the same protocol to examine male players. I have some amendments I would urge you to consider before recommending publication.

Dear Reviewer 2, thank you for your kind comments. We have considered your recommendations and made corrections in the manuscript accordingly.

We agree that it would be interesting to use the same protocol to examine also male players and analyse the interaction of the examined factors with the selection in both genders separately. In our previous article within this project (Tróznai et al., 2021), we examined the prevalence of the RAEs in this type of selection and found significant RAEs in both male and female players. In future research it is our intention to examine the selection in more details including also male athletes.

Tróznai Zs, Utczás K, Pápai J, Négele Z, Juhász I, Szabó T, Petridis L. Talent Selection Based on Sport-Specific Tasks Is Affected by the Relative Age Effects among Adolescent Handball Players. J Environ Public Health. 2021;18(21):11418. [doi:10.3390/ijerph182111418](https://doi.org/10.3390/ijerph182111418)

Title
Consider to remove or revise: „...based on only sport-specific criteria”; from the title.

We removed “sport-specific criteria” from the title as suggested.

Abstract
Be consistent in how your present the likelihood of being selected: Sometimes you use chance (percentages) and sometimes odds, making it a bit confusing to read.

Thank you for this comment. In the revised manuscript we used the terms likelihood or chances instead of the odds to express more precisely the influence (in percentage) of the examined factors on the selection. Hopefully, it is more accurate this way.

Introduction
Add a paragraph that explicitly explains the relationship between relative age effects and maturation. See the work of Liam Sweeney and colleagues for inspiration.

In accordance also with the comments of the first reviewer we have revised the introduction to better clarify the concepts of RAE and maturity and emphasize that they constitute distinct constructs in youth sports.

Context
In your methodology, add a paragraph outlining the context of Hungarian women’s handball and how youth handball is organized (include information about e.g. the cultural significance of handball, number of participants, organized within clubs and/or school sport, requirements for coach certifications, and the level of professionalization in youth sports).

We added a new paragraph in "materials and methods" to present the context of the Hungarian female handball (Lines 115-128) The description briefly presents the responsibilities of the Federation, coaching education system and training and competition structure.

Discussion
Add a paragraph discussing how your findings holds practical significance for policy and practice in talent identification and selection.

We have added a short paragraph under conclusion (Line 410-416) to point out some practical applications based on the results of this study, possible directions to be considered for policy makers and some suggestions for future studies.

Minor issues:
L124 Change „measured players”; to „cohort”;

Corrected accordingly.

L130 Please specify. I reckon this was the university ethics committee?

Yes, ethical approval was received by the Ethics Committee of the University, we added this information in Lines 112-113.

L133 In the methods section, you have used the local, county, and regional level as descriptors. Here, you use different terms. Please revise for consistency.

We have revised the description of the selection program using the terms county stage, regional stage, and national stage throughout the entire manuscript including also within figure 1 and in under methods/selection program.

L224 See my above comment.

This sentence was removed to the statistical analysis paragraph according to Reviewer 1 comments. Based on the above comment, we tried to be consistent in the terms describing the different selection stages, we refer to this as basic stage in the revised manuscript (Ln220-222).

L315 Change „there was a study”; to „we are familiar with one study”;.

Corrected accordingly.

L339 It is unclear who „elite players”; and „the entire sample”; refer to here.

We have revised this part to more accurately describe the entire cohort of all measured players (selected and not-selected) and those players who reached the national (last) selection stage. We consider the national stage of the selection as the elite level of the examined selection program and the players reaching this stage as the elite players in this age group.

L353-358 Grammar off. Please revise.

We revised this paragraph rephrasing the conclusions to better reflect the results of the study and to correct any grammatical issues. Hopefully, it is clearer now.
