# Supplementary material for: The interaction of relative age with maturation and body size in female handball talent selection
Source: PLoS One. 2024 Oct 21;19(10):e0298560. doi: 10.1371/journal.pone.0298560 (PMC11493251; doi:10.1371/journal.pone.0298560)
Supplement: Attachment — Submitted filename: Response to Reviewers.docx [file pone.0298560.s005.docx]

Once again, we would like to thank the reviewer for the time and for the valuable comments. Please find our responses in red following all previous comments.

**Specific and general comments (by section)**

**MATERIAL AND METHODS**

*The Selection Program:*

Regarding the talent programme based on only sport-specific tasks, I have three concerns: (i) given that this type of specific tasks are not carried out from phase 1 to phase 2, could it cause a selection bias? It may be that the coach's recommendation and the results of these tests are not the same so that players who are not potential talents could enter the system or vice versa according to the variables of analysis;

First comment: indeed, the reviewer is right that there may be a selection bias between the first and the following stages due to the different selection criteria. The structure of the selection program was designed and decided by the Hungarian Handball federation, this same structure has been implemented for the last 8 years with 8 selection programs being completed in this form. Since we could not manipulate the selection, we attempted to interpret the results according to the applied criteria, we discuss the issue of coaches’ recommendation in the discussion under (Line 346-357) Of interest is to understand any possible connection between the examined factors and the selection even when selection is based on the subjective evaluation of team coaches.

In my opinion, the Hungarian handball talent identification system might have a weakness in this respect. Regarding the manuscript, please do not only discuss this fact in the discussion but add it as an external limitation of the study in the corresponding section after the Discussion. If you consider it, please let those responsible for the recruitment of young players in the Hungarian Handball Federation know.

We added this thought as a limitation after the discussion under lines 407-409 (in the revised manuscript) or under lines 347-350 in the original manuscript. We are in continuous contact with the Handball Federation to share the analysis of our results and communicate our concerns and experiences. Hopefully, this may have a positive impact on the following selection programs.

(iii) are the tests and scores pre-validated or are they ad hoc tasks that you consider valid to assess handball performance?

Third comment: again, we agree with the reviewer regarding the validity of the selection tasks. As mentioned many times, the selection tasks were designed by experts and federation coaches of the Handball Federation. It was out of our competence to evaluate these tasks, although we agree that a separate validation study would be useful, we are more than pleased to undertake this commitment in future research.

I thank you for your willingness to solve or intervene on the tests to be carried out in order to improve the selection process of young players in a country where handball is so important. However, researchers should be concerned about what to analyse and where to analyse it. Placing research in national sport talent programmes is essential to advance the science in this area, however, examining poorly designed (external) processes could reduce the quality of the study. In the case of the manuscript, please add this limitation as stated in the previous point.

Similarly to the previous comment we also added this as limitation under lines 405-407 (in the revised manuscript) or under lines 347-350 in the original manuscript.

**DISCUSSION**

**GENERAL COMMENT:** The Discussion presents problems, some of them already discussed in the Introduction. On the one hand, there is still no distinction between RAE and maturity status as two different constructs. On the other hand, the references used are not valid in some cases and in parts of this section (i.e., lines 262-282) there is no reference that supports the results or explanatory arguments.

While keeping the structure of the discussion, in the revised manuscript we tried to better discriminate the two factors (maturation and relative age) interpreting the results separately. Were pointed, we added new references to support our statements (e.g. Ln315) or replaced some to be more specific (e.g. Ln350).

The second problem of the Discussion persists, in my opinion. This section should be used to briefly state the main results, arguing the appropriate explanations for them and making them similar to/differentiating them from other results of other studies. However, in these lines (262-282) the authors only make a presentation of results already evaluated in the previous section ‘Results’. Please add to the literature.

On the other hand, I would like to ask for more work to be done on the rebuttal letter with regard to the line numbers where the corrections are made. They do not match in any case and it is extremely difficult to check them a second time.

Thank you for this comment. We revised this paragraph trying to place our results in the literature briefly mentioning the outcomes that have been reported in previous research. The revised paragraph is now under lines 308-332 or under lines 260-285 in the original manuscript. The following citations have been added in the revised manuscript.

# 1 Schorer J, Wattie N, Baker JR. A New Dimension to Relative Age Effects: Constant Year Effects in German Youth Handball. PLoS ONE. 2013;8(4): e60336. <https://doi.org/10.1371/journal.pone.0060336>

# 2 Wrang CM, Rossing NN, Diernæs RM, Hansen CG, Dalgaard-Hansen C, Karbing DS. Relative Age Effect and the Re-Selection of Danish Male Handball Players for National Teams. J Hum Kinet. 2018;63(1): 33-41. doi:10.2478/hukin-2018-0004

# 3 Gil SM, Badiola A, Bidaurrazaga-Letona I, Zabala-Lili J, Gravina L, Santos-Concejero J, Lekue JA, Granados C. Relationship between the relative age effect and anthropometry, maturity and performance in young soccer players. J Sports Sci. 2014;32(5): 479-486. [doi:10.1080/02640414.2013.832355](https://doi.org/10.1080/02640414.2013.832355)

# 4 Müller L, Gehmaier J, Gonaus, C, Raschner C, Müller E. Maturity status strongly influences the relative age effect in international elite under-9 soccer. J Sport Sci Med, 2018;17(2): 216-222.

# We apologize for the confusion in line numbers. After the first revision we included line numbers according to the revised manuscript and not according to the original manuscript. It is true that due to the changes in the revised manuscript, the line numbers do not match. In our current response we included the line numbers as they appear after the second revision as also according to the original manuscript for easier follow up. Hopefully, this helps.
