# Supplementary material for: The interaction of relative age with maturation and body size in female handball talent selection
Source: PLoS One. 2024 Oct 21;19(10):e0298560. doi: 10.1371/journal.pone.0298560 (PMC11493251; doi:10.1371/journal.pone.0298560)
Supplement: Attachment — Submitted filename: Response to Reviewers.docx [file pone.0298560.s006.docx]

**RESPONSE TO REVIEWERS**

Once again, we would like to thank the reviewer for the time and for the valuable comments. Please find our responses in red.

Review Comments to the Author

Reviewer #1: The authors have made a great effort to make all the modifications and changes proposed by the reviewer. I now consider the article ready for publication. Therefore, I would like to congratulate each and every one of the authors of the article.

Thank you very much for your kind words.

Reviewer #2: Thank you for the opportunity to read your manuscript, which I found to be both novel and thoroughly conducted. Overall, it makes a strong contribution to the research literature. However, I find the discussion section somewhat restricted. It would benefit from more courageous argumentation and suggestions for policy and practice. It is not entirely clear how increased monitoring of RAEs and biological maturation would lead to improved talent identification and development, though it might result in fairer selections. I recommend the authors read the recent contribution by Sweeney et al. (2023) for further insights (full reference provided below).

We would like to thank the reviewer for his kind words and for the valuable comments. We revised and expanded in more details the last paragraph under conclusions where recommendations for policy are discussed taking also into consideration the paper recommended by the reviewer (Sweeney et al., 2023). As indicated in the revised paragraph, we consider it important that the selection is not limited only to one participation, but the players have the chance to participate in the talent selection process in multiple occasions by rotating the cut-off dates, so the players may experience being in the older and younger age groups potentially counteracting in this way the significant underrepresentation of players born in the second year. However, it should be noted that the efficiency of the possible intervention strategies has yet to be examined, currently there is not enough evidence to support the use of a specific intervention over another one.

Apart from these points, I have only a few minor issues before recommending publication. Congratulations to the authors for their efforts.

Thank you.

Abstract
- Lines 32-33: To improve coherence, consider using either percentages or multiplicatives consistently. Is there a specific reason for presenting the data as it is? This also applies to the results section.

We corrected multiplicative to percentages in the abstract and in the results. We found the use of percentage easier to describe the influence of the examined variables on the selection.

Introduction
- Line 59: Change “participation” to “performance”.

corrected accordingly
- Line 72: Elaborate on the details of this study [33].

We rephrased this sentence slightly expanding on the details of the cited paper. Also, we added an additional reference (Bjørndal et al., 2018), where the authors reported no RAE among selected players at the senior age group.

Conclusion
- Lines 418-420: This sentence is confusing. Please revise for better clarity.

We rephrased this sentence, hopefully is clearer now.

As recommended, the following paper has been included in the revised manuscript:

Sweeney, L., Taylor, J., & Macnamara, Á. (2023). Push and Pull Factors: Contextualising Biological Maturation and Relative Age in Talent Development Systems. Children, 10(1), 130.[https://doi.org/10.3390/children10010130](<https://doi.org/10.3390/children10010130>)
